# Disruption of *TgPHIL1* Alters Specific Parameters of *Toxoplasma gondii* Motility Measured in a Quantitative, Three-Dimensional Live Motility Assay

**DOI:** 10.1371/journal.pone.0085763

**Published:** 2014-01-29

**Authors:** Jacqueline M. Leung, Mark A. Rould, Christoph Konradt, Christopher A. Hunter, Gary E. Ward

**Affiliations:** 1 Department of Microbiology and Molecular Genetics, University of Vermont, Burlington, Vermont, United States of America; 2 Program in Cellular, Molecular and Biomedical Sciences, University of Vermont, Burlington, Vermont, United States of America; 3 Department of Molecular Physiology and Biophysics, University of Vermont, Burlington, Vermont, United States of America; 4 Department of Pathobiology, School of Veterinary Medicine, University of Pennsylvania, Philadelphia, Pennsylvania, United States of America; University of Kentucky, United States of America

## Abstract

*T. gondii* uses substrate-dependent gliding motility to invade cells of its hosts, egress from these cells at the end of its lytic cycle and disseminate through the host organism during infection. The ability of the parasite to move is therefore critical for its virulence. *T. gondii* engages in three distinct types of gliding motility on coated two-dimensional surfaces: twirling, circular gliding and helical gliding. We show here that motility in a three-dimensional Matrigel-based environment is strikingly different, in that all parasites move in irregular corkscrew-like trajectories. Methods developed for quantitative analysis of motility parameters along the smoothed trajectories demonstrate a complex but periodic pattern of motility with mean and maximum velocities of 0.58±0.07 µm/s and 2.01±0.17 µm/s, respectively. To test how a change in the parasite's crescent shape might affect trajectory parameters, we compared the motility of Δ*phil1* parasites, which are shorter and wider than wild type, to the corresponding parental and complemented lines. Although comparable percentages of parasites were moving for all three lines, the Δ*phil1* mutant exhibited significantly decreased trajectory lengths and mean and maximum velocities compared to the parental parasite line. These effects were either partially or fully restored upon complementation of the Δ*phil1* mutant. These results show that alterations in morphology may have a significant impact on *T. gondii* motility in an extracellular matrix-like environment, provide a possible explanation for the decreased fitness of Δ*phil1* parasites *in vivo*, and demonstrate the utility of the quantitative three-dimensional assay for studying parasite motility.

## Introduction

The phylum Apicomplexa includes a number of protozoan parasites that are of major veterinary and medical importance, including *Plasmodium* spp., the causative agents of malaria, and *Toxoplasma gondii*, which infects approximately one-third of the world's population and can cause life-threatening complications during pregnancy and in immunocompromised individuals. *T. gondii* serves as a powerful model organism for studying conserved aspects of apicomplexan parasite biology since it can be readily cultured *in vitro*, it is highly amenable to molecular genetic manipulation, and well-established mouse models exist for *in vivo* studies [1]–[4].

As with the other parasites in the phylum, motility is critical for the virulence of *T. gondii*. The asexual, fast-replicating and invasive stage (*i.e.*, the tachyzoite) uses a unique form of gliding motility to travel to and egress from the host cells it invades, migrate across biological barriers and spread through tissues of the infected host [5], [6]. Gliding motility is substrate-dependent and involves neither force-generating appendages such as cilia and flagella, nor significant changes in parasite shape. Instead, motility is powered by a myosin motor complex that lies just beneath the parasite plasma membrane [7], [8]. The exact spatial and structural organization that enables the myosin motor to drive productive parasite motility is not yet fully understood, although a variety of small molecules [9]–[16] and mutations in motor complex components [4], [17]–[19] that affect parasite motility have been identified and will be useful for dissecting the underlying mechanisms.

*T. gondii* motility is most often studied on protein-coated glass coverslips. As the parasites glide along the surface of the substrate, they leave behind “slime trails” containing the major GPI-linked surface protein, TgSAG1, which can be stained and visualized by immunofluorescence microscopy [9], [20]. By live videomicroscopy, parasites exhibit three types of motility on such two-dimensional (2D) surfaces: twirling, where the parasite is upright on its posterior end and rotates clockwise; circular gliding, where the parasite lies on its side and moves in counterclockwise circles; and helical gliding, where the parasite glides in a clockwise arc while rotating 180° about its curved long axis, then lifts its apical end off the surface and flips back onto its left side to start another cycle [11]. The distinct crescent shape and cytoskeleton of the parasite are likely to play a role in the curved trajectories of both circular and helical gliding [11], [19], [21]. How the three modes of motility observed in 2D translate to movement in the three-dimensional (3D) environment the parasite encounters *in vivo* is unclear.

In this study, we describe the motility of *T. gondii* in an *ex vivo* murine earflap model, and present a semi-automated assay that quantitatively measures parameters of parasite motility within a controlled, 3D extracellular matrix-like environment (Matrigel). In marked contrast to what is seen in 2D, all tachyzoites were found to move in irregular corkscrew-like trajectories in 3D. To test whether parasite shape influences their motility in Matrigel, we quantified and compared the trajectory parameters of wild-type parasites and parasites lacking the cytoskeleton-associated protein, TgPHIL1. *TgPHIL1* knockout (Δ*phil1*) parasites are shorter and wider than wild type, and markedly less fit in a mouse model of infection. We show here that they also move more slowly and travel shorter distances in the Matrigel assay, suggesting that parasite shape may indeed play a role in determining how *T. gondii* tachyzoites move within an extracellular matrix-like environment.

## Materials and Methods

### Ethics Statement

All animal studies were carried out in compliance with the guidelines of the Institutional Animal Care and Use Committee (IACUC) of the University of Pennsylvania and in accordance with the recommendations in the Guide for the Care and Use of Laboratory Animals of the National Institutes of Health. The animal protocol was approved by the Institutional Animal Care and Use Committee (IACUC) of the University of Pennsylvania, Philadelphia.

### Parasite culture

Wild-type (RH) strain, RHΔ*phil1*, RHΔ*phil1*/*TgPHIL1-C5* (Comp) and RH-OVA-tdTomato *T. gondii* tachyzoites [22], [23] were maintained by serial passage in confluent primary human foreskin fibroblast (HFF) (ATCC CRL-1634) monolayers in Dulbecco's Modified Eagle's Medium (DMEM), supplemented with 10% (v/v) heat-inactivated fetal bovine serum (FBS) and 10 mM HEPES pH 7.0, as previously described [24]. The medium was changed to DMEM supplemented with 1% (v/v) heat-inactivated FBS and 10 mM HEPES pH 7.0 just prior to infecting the confluent monolayers with parasites.

### *ex vivo* imaging

C57BL/6 mice (Jackson Laboratories, Bar Harbor, ME) were anaesthetized by intraperitoneal injection of Ketamine/Xylazine. Ear hair was removed with a hair removal solution (Nair, Princeton, NJ). 1–2×10^5^ freshly egressed RH-OVA-tdTomato tachyzoites in 1 µL PBS were injected intradermally using a 26-gauge Hamilton syringe. Five min post injection the mouse was euthanized in a CO_2_ chamber and the ear was removed and placed in a temperature-controlled imaging chamber (Warner Instruments, Hamden, CT), and embedded in 1.2% (w/v) low-melting agarose (Sigma-Aldrich, St. Louis, MO). Tissue was perfused with RPMI complete (RPMI plus 10% FBS, 1% penicillin/streptomycin, 1% glutamine, 1% HEPES, 1% nonessential amino acids, and 0.1% beta-mercaptoethanol), perfused with 95% oxygen and 5% carbon dioxide, and maintained at 37°C. *ex vivo* imaging was performed using a Leica SP5 two-photon microscope equipped with a picosecond laser (Coherent Chameleon; 720 nm–1020 nm) and tunable external detectors that allow simultaneous detection of emissions of different wavelengths and second harmonic signals (SHG). tdTomato was excited using a laser light of 900 nm. The LAS-AF software (Leica) was used for image acquisition.

### Pitta chamber construction and parasite preparation for 3D motility assays

To construct imaging (“Pitta”) chambers, washed 22×22 mm borosilicate glass coverslips were adhered to washed 24×50 mm borosilicate glass coverslips with strips of double-sided tape (Scotch 3M, St. Paul, MN) spaced 3 mm apart, for an internal volume of ∼10 µL.

Parasites were harvested by syringe release of infected HFF monolayers through a 27-gauge needle, and then filtered through a 3 µm Nuclepore filter (Whatman, Piscataway, NJ), centrifuged at 1,000× *g* for 4 min, washed and resuspended at a concentration of 1–2×10^8^ parasites/mL in 3D Motility Media (1× Minimum Essential Medium lacking sodium bicarbonate, 1% (v/v) FBS, 10 mM HEPES pH 7.0 and 10 mM GlutaMAX L-alanyl-L-glutamine dipeptide) supplemented with 0.3 mg/mL Hoechst 33342 (H33342). Matrigel (BD Biosciences, San Jose, CA) was kept on ice to prevent polymerization prior to mixing with the parasite suspension and 3D Motility Media in a ratio of 3∶1∶3 by volume, respectively. Pitta chambers were perfused with ∼10 µL of this parasite/Matrigel suspension (final H33342 concentration ∼43 µg/mL) and incubated at 27°C for 7 min on a ThermoPlate (Tokai Hit, Shizuoka-ken, Japan). The chambers were then placed in a NanoScanZ piezo Z stage insert (Prior Scientific, Rockland, MA) in a custom-built, preheated microscope enclosure (UVM Instrumentation and Model Facility, Burlington, VT), and incubated at 35±0.2°C for 2 min to allow for temperature equilibration and completion of Matrigel polymerization prior to data acquisition. The room housing the microscope was also heated to the same temperature (35±1°C) to maintain temperature equilibration and minimize thermal convection currents within the microscope enclosure. The kinetics of Matrigel polymerization varied slightly depending on the reagent lot number, but were generally consistent under these empirically optimized conditions.

For live trail deposition in Matrigel, tachyzoites were harvested as described above, and incubated at 25°C for 15 min in 3D Motility Media with a 1∶25 (v/v) dilution of anti-TgSAG1 antibody (Argene, Shirley, NY) directly conjugated to Alexa Fluor 488 (Invitrogen) as previously described [9]. Labelled parasites were washed once with 3D Motility Media, then mixed with 3D Motility Media and chilled Matrigel, perfused into Pitta chambers and processed as for H33342-labelled samples.

Heat-killed tachyzoite preparations were prepared by incubating at 56°C for 30 min as described previously [25]. Cytochalasin D was dissolved to a concentration of 1 mM in high quality DMSO and stored in the dark at −20°C. This stock solution was diluted to a final compound concentration of 0.2 µM in 3D Motility Media supplemented with H33342 for parasite treatment. The suspension was then incubated at 37°C for 15 min to inhibit parasite motility as described previously [26], [27], prior to mixing with Matrigel and imaging.

### Imaging and data acquisition

Fluorescent parasite nuclei were imaged using a 20× PlanApo λ objective (NA = 0.75) on a preheated Nikon Eclipse TE300 epifluorescence microscope with neutral density (ND) 4, ND 8 (Nikon Instruments, Melville, NY) and ND OD = 1.0 filters (Thorlabs, Newton, NJ) installed in tandem. Reference slides containing images with a defined chirality were used to confirm the correct orientation of the microscope and interpretation of trajectory handedness with the analysis software.

Time-lapse stacks were captured using an iXon 885 EMCCD camera (Andor Technology, Belfast, Ireland) cooled to −70°C and driven by NIS Elements v. 3.20 software (Nikon Instruments, Melville, NY). The camera was set to frame transfer sensor mode, with a vertical pixel shift speed of 1.0 µs, vertical clock voltage amplitude of +1, readout speed of 35 MHz, conversion gain of 3.8×, EM gain setting of 3 and 2×2 binning. The z-slices were imaged with an exposure time of 16 ms; stacks consisted of 41 z-slices spaced 1 µm apart for a total x, y, z, t imaging volume of 402 µm×401 µm×40 µm for 67 stacks in 60 s. All experiments were completed within 80 min of harvesting the parasites, and control (*i.e.*, untreated) samples were assayed both at the very beginning and end of each experiment to ensure imaging and parasite conditions remained constant. To observe live trail deposition, tachyzoites labelled with Alexa Fluor 488-conjugated anti-TgSAG1 were imaged as described above but with a single ND filter (ND 8) installed, and with a camera EM gain setting of 10.

### Tracking

Datasets were exported from NIS Elements and read directly in Imaris ×64 v. 7.6.1 (Bitplane AG, Zurich, Switzerland). Using the ImarisTrack module, parasites were tracked within a region of interest (ROI) that was cropped 1 µm from each of the original x, y and z dimensions to avoid tracking artifacts associated with objects close to the border. Background object subtraction and elliptic spot detection in the z-dimension were enabled, with the estimated x, y diameters set to 4.0 µm and the estimated z diameter set to 8.0 µm. A filter was applied to the datasets for tracks that came within 0.4 µm of a ROI border, as well as a filter to exclude all tracks with durations of less than ∼2 s, to further avoid tracking artifacts as mentioned above. An autoregressive motion tracking algorithm was applied with a maximum distance of 12.0 µm and a maximum gap size of 1. Translational and rotational drift correction were employed where necessary (*e.g.*, a dataset where stationary parasites all have the same displacement vector) and datasets were manually inspected following drift correction to ensure the algorithm had been executed appropriately. Positional coordinates were then exported in comma-separated values (.csv) format for further processing and analysis using the Bugs software suite.

### Bugs software processing and analysis

Subsequent processing and analysis of the acquired trackpoints for each organism were accomplished by a custom software suite we developed specifically for this purpose. A key tenet of the software is that the user be able to graphically and quantitatively verify each step in the process. To that end, the digitized track data were converted to and maintained at all times in a format (PDB, Brookhaven Protein Data Bank format; www.pdb.org) that allows direct visualization and analysis of all the organisms' discrete trackpoints (or smoothed trajectories, described below) with molecular graphics software such as PyMOL [28], [29]. For further quantitative analysis and plotting, derived values such as velocity, curvature and torsion were written to .csv format files for use with spreadsheet or plotting software. The “Bugs” software suite is written as portable Fortran modules linked together by shell scripts, and is available by download by SFTP from http://sourceforge.net/projects/bugs/. For questions about the software, contact mrould@uvm.edu.

Parasites with a total trajectory displacement (simple distance from first to last trackpoint) of greater than 2 µm were considered moving based on analysis of a heat-killed parasite preparation (see Results); these trajectories were subject to Fourier smoothing and calculation of parameters as discussed below. Parasites with a total trajectory displacement of 2 µm or less were considered stationary and excluded from further analysis.

### Fourier smoothing

The rather sparse temporal sampling of the position of each organism, coupled with positional error in those coordinates necessitated some form of smoothing and interpolation be applied to the trackpoint coordinates. A simple, modified Fourier fit to each organism's entire set of trackpoints performed the best of several approaches tested. We evaluated these approaches using an “even-odd” cross-validation criterion in which the even-numbered trackpoints were used in the fit, and the cross-validated residual discrepancy was determined between the observed and fit coordinates of the odd-numbered trackpoints (which were not used to determine the parameters of that fit). Similarly, the odd-numbered trackpoints were used in the fit and the fit discrepancy measured with the even-numbered trackpoints. The total cross-validated discrepancy was calculated as the square-root of the sum of these two squared discrepancies. This form of cross-validation mimics the effects of a data acquisition rate that is half of the true rate, and quantifies how well we would be able to predict the “missing” data in that case. The three spatial coordinates of each organism's trajectory were fit independently as a function of time (x(t), y(t), z(t)) using singular value decomposition [30]. The fit parameters were the coefficients of the standard Fourier series, 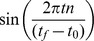 and 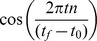, where n runs from 0 to the order, m, of the Fourier series, supplemented by an additional linear term, time. The latter extra term allowed better fits using a lower Fourier order (and hence requiring fewer free parameters) by permitting compensation for any difference in the first, x(t**_0_**), and last, x(t**_f_**), values (since the Fourier series is intrinsically periodic). The target function was the sum of the squared discrepancies between the fit values x(t**_i_**) and the observed values X**_obs_**(t**_i_**) of the coordinates. Optimal Fourier order, m, for the fit for each organism's trajectory was determined using even-odd cross-validation. Fourier fits were applied as long as there were a sufficient number of trackpoints (≥12) in a given trajectory to do so. The number of trackpoints was not necessarily correlated to trajectory length; consequently, a subset of trajectories from moving parasites was considered “unfittable” and excluded from subsequent analysis.

### Trajectory analysis

Coordinates of the smoothed trajectory at a given time are calculated by evaluation of the Fourier series (supplemented by the linear time term) with coefficients as fit above. A trajectory sampled at regular intervals of arc length is readily calculated by computational integration of arc length over time. Analytically-derived expressions for the first, **s**′, second, **s**″, and third, **s**′″, time derivatives of the 3D Fourier series (including the time term) are used to calculate the instantaneous velocity (v), curvature (κ) and torsion (τ) at any given point along the trajectory via the equations [31]:
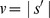


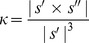


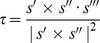



The total trajectory length for a given organism is the (computationally) integrated path length (contour length) along the smoothed trajectory from the first to last trackpoint. The total displacement is the simple distance (“as the crow flies”) from the first to last trackpoint for an organism. The last trackpoint is derived from the last z-stack acquired in the 60 s dataset, or the last z-stack acquired before the fluorescent parasite nucleus moves out of the volume being imaged.

Discrete measurements of velocity were calculated as the simple distance between two consecutive trackpoints sampled at regular (0.5 µm) intervals of pathlength along the Fourier-smoothed trajectory, divided by the time interval between those points. The maximum velocity corresponds to the highest value for velocity observed along the Fourier-smoothed trajectory.

### Statistical analysis

Values for motility parameters were exported from the Bugs software suite in .csv format, imported into Microsoft Excel for data compilation, and graphed and analyzed using GraphPad Prism v. 6.01 (La Jolla, CA). Parameters calculated from motility assays and graphed as scatter plots were analyzed using paired (RH *vs.* Δ*phil1* and Δ*phil1 vs.* Comp) and unpaired (RH *vs.* Comp) Student's t-test; frequency distributions were analyzed by performing a Kolmogorov-Smirnov test. Statistical significance between samples is indicated with asterisks. Results shown are the mean ± standard deviation of four to five independent experiments, each performed in either triplicate or quadruplicate.

## Results

### *T. gondii* move with corkscrew-like trajectories in a three-dimensional environment

**Figure 1:**
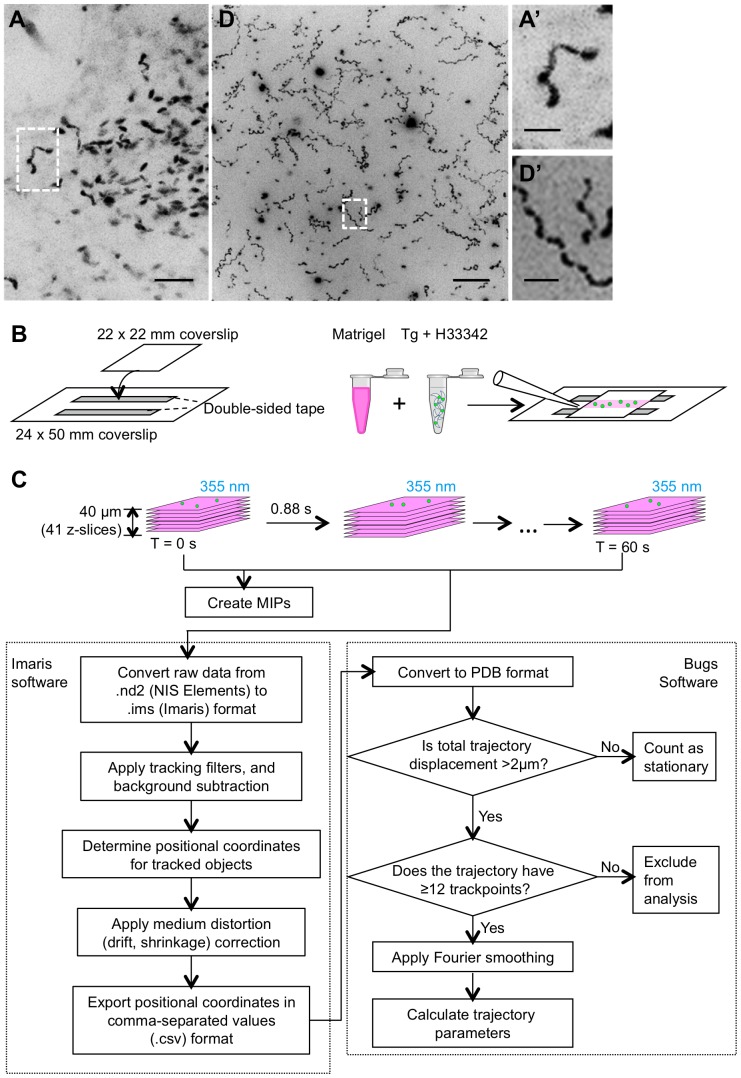
*T. gondii* moves in a corkscrew-like manner in three dimensions. (**A**) RH strain *T. gondii* tachyzoites expressing a tandem tomato fluorescence cassette (RH-OVA-tdTomato) were injected into a mouse earflap and imaged by two-photon laser scanning microscopy. A maximum intensity projection (MIP) shows parasites that move in a corkscrew-like fashion (*e.g.*, dashed white box). Scale bar = 29 µm. See Video S1 for the corresponding movie. (**A'**) Higher magnification view of dashed white box in (A); scale bar = 12 µm. (**B**) Assembly and dimensions of the imaging (“Pitta”) chamber. Coverslips were assembled using double-sided tape, and perfused with a 1∶3∶3 mixture of parasites treated with Hoechst 33342, 3D motility media and chilled Matrigel, respectively. (**C**) Pitta chambers were incubated at 27°C for 7 min, followed by 2 min equilibration in the preheated 35°C microscope enclosure. Fluorescent parasite nuclei were imaged for 60 s in a 402 µm×401 µm×40 µm volume (approximately 67 z-stacks, with 41 z-slices captured every 0.88 s) by time-lapse fluorescence videomicroscopy. Datasets were visualized during acquisition by generating MIPs, tracked using the Imaris software, and then analyzed using the Bugs software as indicated in the workflow. (**D**) A MIP showing that parasites also move in corkscrew-like trajectories in Matrigel. Scale bar = 50 µm. See Video S3 for the corresponding movie. (**D'**) Higher magnification view of dashed white box in (D); scale bar = 10 µm. The colour scheme for all MIPs was inverted for better visualization of parasite trajectories.

To investigate how *T. gondii* tachyzoites move in animal tissue, we used a model of *T. gondii* infection where live tachyzoites expressing a tandem Tomato fluorescence reporter (RH-OVA-tdTomato) are injected into the mouse earflap and imaged by two-photon laser scanning microscopy [32]. Unlike the twirling, circular gliding and helical gliding observed in 2D motility assays, tachyzoites in the earflap appeared to undergo a form of corkscrew-like motility (Figures 1A and 1A', and Video S1). This suggested that the standard 2D motility assay may not be a completely accurate reflection of parasite motility within host tissues.

We sought to study this behaviour with larger numbers of parasites, and in a more defined and controlled environment. To accomplish this, an *in vitro* assay was developed where imaging (“Pitta”) chambers were perfused with parasites resuspended in Matrigel, a soluble basement membrane preparation that has been widely used as a matrix to study cell migratory behaviour in 3D [33]–[35]. Since the parasites were pre-labelled with Hoechst 33342, a fluorescent nucleic acid stain, the positions of the fluorescent parasite nuclei could be readily imaged in the chamber volume by time-lapse fluorescence videomicroscopy (Figures 1B and 1C). Analysis of the datasets revealed that all of the parasites deposited TgSAG1-containing trails (Video S2) as they moved in left-handed, corkscrew-like trajectories through the Matrigel (see maximum intensity projection (MIP) in Figures 1D and 1D', and Video S3). These data demonstrate that the trajectories of *T. gondii* tachyzoites are strikingly different in a 3D environment compared to the types of motility observed on a coated glass coverslip, and that it is possible to reconstitute this behaviour *in vitro* using a Matrigel-based system.

### Quantification of parameters of 3D trajectories

To further characterize and quantify the parameters of this form of motility, we developed semi-automated methods for tracking the positional coordinates of the parasites and reconstructing their trajectories for detailed analysis. Two types of “stationary” parasite datasets were generated to estimate an overall error due to Brownian motion and tracking error from the analysis software. Out of several criteria tested, the total trajectory displacement (*i.e.*, the shortest distance from the initial to the final position of the parasite) performed the best in terms of distinguishing between moving and stationary parasites (data not shown). In a heat-killed preparation, 97.6% of the parasite trajectories had a displacement of 2 µm or less (Figures S1A and S1B), with mistracking artifacts (*e.g.*, trajectories connecting two parasites that were clearly stationary) accounting for the remaining 2.4% (data not shown). Similar results were obtained when parasites were treated with 0.2 µM cytochalasin D (Figures S1C and S1D), a concentration previously described to inhibit parasite motility in 2D assays [26], [27].

**Figure 2:**
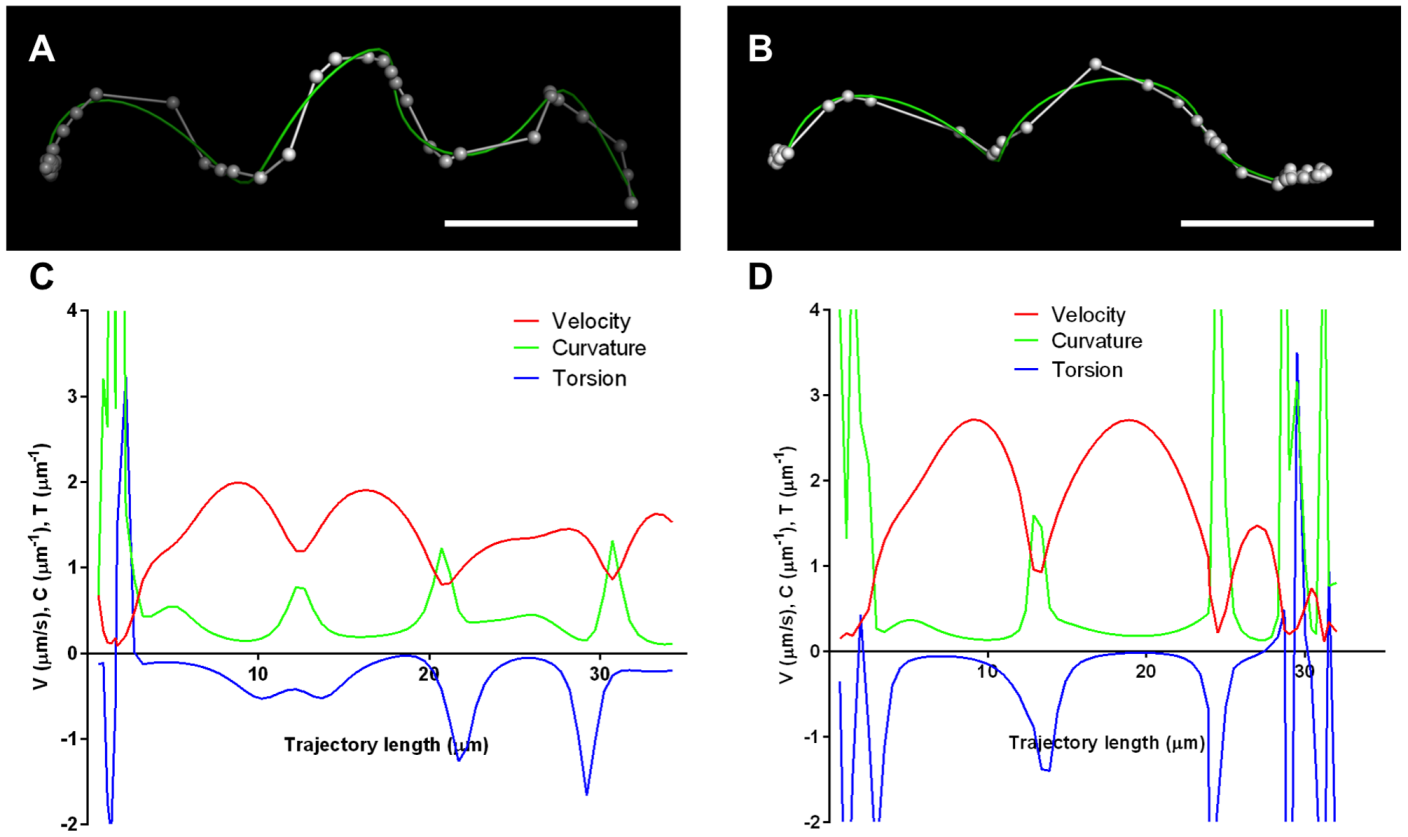
Visualization and analysis of two representative 3D trajectories of parasites in Matrigel. (**A**) and (**B**) Positional coordinates for two representative wild-type (RH) parasite trajectories are visualized as connected, discrete trackpoints (white), and overlaid with the trajectory after smoothing (green). Scale bar = 7 µm. (**C**) and (**D**) Plots of velocity (red), curvature (green) and torsion (blue) values along the length of the parasite trajectories shown in panels A and B, respectively. The curvature and torsion values would be constant through time for a regular helix; the variation in these measurements along the parasite trajectories shows that they move in irregular-shaped yet periodically fluctuating corkscrews.

When the trajectories were smoothed by applying a modified Fourier fit (Figures 1C, 2A, 2B, and S2, S3, S4, S5), it was apparent that the parasite's velocity along the trajectory oscillated between minima and maxima, reflecting bursts of speed as the parasites moved through the Matrigel (Figures 2C and 2D). Furthermore, a complex and periodic relationship between velocity, curvature (*i.e.*, degree to which the trajectory bends within a plane; see Figure S6A and S6B) and torsion (*i.e.*, degree to which the trajectory twists out of the plane; see Figure S7A and S7B) was evident along the trajectory. The trajectories decreased in curvature and torsion as the parasites accelerated (*i.e.*, as their velocity increased), but once the local maximum velocity was reached, gradual deceleration was associated with progressively increasing curvature and torsion (Figures 2C and 2D). When curvature and torsion reached their maxima, the parasite either repeated the cycle, or paused temporarily for a variable period of time marked by low velocity and rapidly fluctuating curvature and torsion. Note that the torsion values are negative to reflect the left-handed nature of the trajectories, such that increasing torsion is depicted as increasingly negative values on the plots [36]. Since a regular helical trajectory would have constant curvature and torsion values along the length of the helix, the parasite-derived trajectories can best be characterized as periodic but irregular helices.

### Δ*phil1* parasites are less motile in a Matrigel-based environment

To assess how a change in the parasite's crescent morphology could affect motility behaviour, we tested the ability of *TgPHIL1* (*Ph*otosensitized *I*NA-*L*abeled protein *1*) knockout parasites to move in the 3D motility assay. TgPHIL1 is a cytoskeleton-associated protein, the absence of which results in parasites that are shorter and wider than parental wild-type (RH) parasites. Complementation of the Δ*phil1* parasites with a copy of *TgPHIL1* driven by its endogenous promoter (“Comp”) rescues the morphological defect (Figure S8) [22].

**Figure 3:**
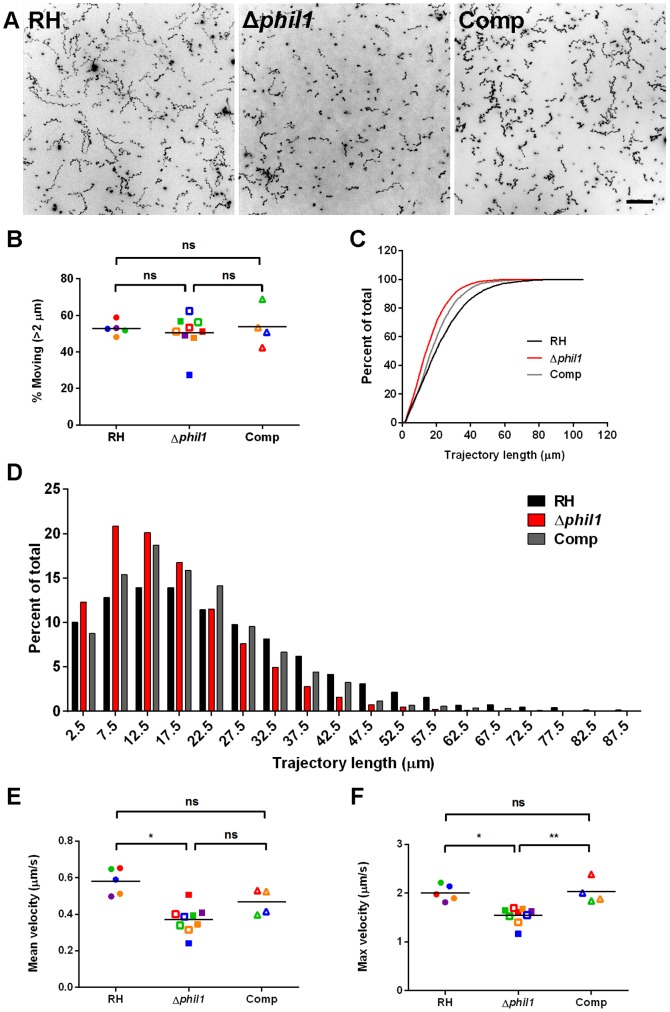
3D motility of the Δ*phil1* parasites. (**A**) MIPs for wild-type (RH), *TgPHIL1* knockout (Δ*phil1*) and complemented (Comp) parasites. Scale bar = 50 µm. The colour scheme for all MIPs was inverted for better visualization of parasite trajectories. The percentage of total parasites moving (**B**) was comparable for the three parasite lines, but the cumulative frequency distribution (**C**) and histogram (**D**) of the smoothed trajectory lengths for RH (black), Δ*phil1* (red) and Comp parasites (grey) reveal that the Δ*phil1* parasites do not move as far as the RH or Comp parasites within the same timeframe (Kolmogorov-Smirnov test, D = 0.199, p<0.0001 and D = 0.114, p<0.0001, respectively). The Δ*phil1* parasites also exhibited significantly decreased mean velocity compared to the RH parasites (**E**) and significantly reduced maximum velocity compared to both RH and Comp parasites (**F**) (paired t-test, significance indicated by asterisks). Closed data points are the results from five independent experiments comparing RH and Δ*phil1* parasites; open data points are the results from four independent experiments comparing Δ*phil1* and Comp parasites. Each of the independent experiments (assigned a different colour in the scatter plot) was performed in either triplicate or quadruplicate. The total number of parasites analyzed was 6,467 for RH, 9,305 for Δ*phil1* and 3,743 for Comp. * p<0.05, ** p<0.001, ns = not significant.

Thousands of trajectories were analyzed (RH n = 6,467, Δ*phil1* n = 9,305 and Comp n = 3,743), and comparable percentages of parasites moved for all three lines (Figures 3A and 3B; summarized in Table 1). However, the Δ*phil1* parasites showed significantly shorter trajectory lengths than the RH parasites from which they were derived (Figures 3A, 3C and 3D; Table 1). The Δ*phil1* parasites also exhibited significantly reduced mean and maximum velocities compared to RH (Figures 3E and 3F; Table 1). These effects were either partially or fully restored in the Comp parasites (Figures 3E and 3F; Table 1), demonstrating that disruption of *TgPHIL1*, which alters parasite morphology, is associated with a significant impact on *T. gondii* motility in Matrigel.

**Table 1:** Summary of 3D motility parameters.

	RH	Δ*phil1*	Comp
n total tracks analyzed	6,467	9,305	3,743
% moving^a^	52.89±3.84%^d^	50.55±9.78%	53.87±11.03%
% fittable moving parasites^b^	85.73±2.71%	89.00±4.08%	92.68±4.26%
median trajectory length (µm)^c^	18.84±2.56	13.94±2.38	18.40±2.80
mean velocity (µm/s)^c^	0.58±0.07	0.37±0.07	0.47±0.07
max velocity (µm/s)^c^	2.01±0.17	1.54±0.17	2.03±0.25

a Percentage of parasites whose trajectories have a total displacement of >2 µm.

b Percentage of parasites with a sufficient number of trackpoints (≥12) to apply a modified Fourier fit.

c Calculated for fittable trajectories from moving parasites only.

d Values expressed are mean ± SD except for trajectory lengths, which are expressed as median ± SD due to their non-Gaussian distribution.

## Discussion

There is a growing interest in developing techniques to track how organisms move through biologically relevant media/substrates, as well as for analyzing the parameters of their movement in a more quantitative way. For instance, digital holographic microscopy was used to characterize the swimming behaviour of dinoflagellates [37], [38], while lensfree, holographic on-chip imaging was performed to analyze human and horse spermatozoa trajectories [39], [40]. In a study that combined aspects of biology and differential geometry, a “finite helix fit” was applied to trajectories of freely swimming organisms such that they could be mathematically expressed and compared using elements of the Frenet-Serret parameters (*i.e.*, velocity, curvature and torsion) [36].

For apicomplexan parasites, efforts to study motility have to date concentrated on quantifying parameters of motility in 2D, and generating tools for semi-automated classification of the types of *T. gondii* tachyzoite and *Plasmodium* sporozoite motility [18], [41], [42]. The motile behaviour of *Plasmodium* sporozoites and ookinetes was further characterized *in vivo* and quantitatively evaluated in Matrigel [34], [43]. In the study reported here, we build upon the previous observations for *T. gondii* tachyzoites by introducing a third physical dimension, and we describe a workflow wherein x, y, z coordinate data are acquired at discrete time intervals using a relatively simple, single-camera setup. Image acquisition is coupled to software for semi-automated tracking and parameter analysis to yield a medium-throughput system where the motility of hundreds of tachyzoites can be simultaneously imaged and analyzed. This provides a quantitative way to determine what specific trajectory parameters are perturbed by mutations or small molecules that alter parasite motility.

We show here that all tachyzoites moving in a mouse earflap or in Matrigel do so in corkscrew-like trajectories. Interestingly, *Plasmodium* sporozoites move in circles on glass, but as they migrate through murine hepatocytes *in vivo* they also adopt a more corkscrew-like pattern [33]. Circular gliding is only one of three types of motility that *T. gondii* exhibits in 2D; tachyzoites also glide helically or twirl [11]. Helical gliding could be the result of a parasite attempting to undergo 3D corkscrews constrained by the 2D surface. Similarly, the clockwise twirling seen in 2D could reflect a parasite that is attempting to move up and away from the substrate in a left-handed corkscrew trajectory, but is tethered to the surface by its posterior end. The mechanism and biological relevance of the counterclockwise circular gliding seen in 2D are less clear, and it will be of interest to determine how this particular mode of motility translates into 3D. For example, parasites lacking the microneme protein TgMIC2 move primarily by circular gliding [18]; these mutants could be analyzed in the assay described here to see how their 3D motility parameters compare to those of the corresponding parental lines. Changes in parasite intracellular calcium levels also appear to be associated with and important for active gliding in 2D [44], [45]. Experiments are currently underway to investigate how these calcium fluxes might correlate with the periodic fluctuations in velocity observed as parasites move in 3D.

TgPHIL1 is a cytoskeleton-associated protein that plays a role in determining parasite shape [22]. Although no significant differences were seen in the invasion or 2D motility of parasites lacking *TgPHIL1*, they were outcompeted by the wild-type and complemented parasites *in vitro*, and appeared to have a selective disadvantage *in vivo* [22]. The 3D Matrigel-based assay revealed that there is in fact a motility defect in the Δ*phil1* parasites: those that were motile moved less far and more slowly than wild type. Since the motility of a parasite is thought to be important for its ability to establish infection and traverse biological barriers [5], a motility defect could be one possible, physiologically relevant explanation for decreased fitness of the Δ*phil1* parasites *in vivo*.

The distinct shape of *T. gondii* is in part defined by the 22 subpellicular microtubules that radiate from the apical polar ring and form a left-handed spiral, terminating two-thirds down the long axis of the parasite [46]. Interestingly, the corkscrews observed in 3D are all left-handed, which is consistent with the left-handed spiral arrangement of the subpellicular microtubules [46], [47]. The polar rings, from which the subpellicular microtubules emanate, do not appear to be tilted in *T. gondii*, unlike what is observed for *Plasmodium* sporozoites and thought to provide a basis for their preferred chirality and directionality of movement [48]. How the proteins of the myosin motor complex are arrayed within the *T. gondii* tachyzoite relative to the microtubule cytoskeleton is an important question to address, if we are to understand and ultimately model parasite motile behaviour.

The morphology of several microorganisms appears to be associated with the ability to move in their respective environmental niches. The shape and curvature of the spirochete *Helicobacter pylori* is thought to be optimally tuned for the human stomach, a high-viscosity habitat; mutants that are rod-shaped or highly curved due to changes in peptidoglycan crosslinking are unable to undergo directional motility in mucin (gel)-like media, and are attenuated in stomach colonization [49], [50]. The curvature of the bacterium could enhance locomotive efficiency by increasing torsion in a high viscosity environment, whereas low viscosity environments such as culture media would promote “slippage” [51]–[53]. *Plasmodium* ookinetes deficient in IMC1h experience a loss of cell rigidity that results in a change in morphology, and move with significantly decreased speeds in Matrigel [34]. Using a Matrigel-based assay similar to the one described here, an association was also recently shown between the shape and helical trajectories of *Plasmodium* ookinetes in Matrigel (J. Baum, personal communication). Taken together, these observations suggest that apicomplexan parasites may share a structural basis for their corkscrew-like trajectories in a 3D environment. It will be of interest to test other *T. gondii* shape mutants, such as the longer and thinner His28Gln tubulin mutant [54], and matrices with different stiffnesses/viscosities to determine what combination(s) of shape and medium stiffness provide for optimal tachyzoite motility. The Matrigel-based assay presented here generates robust, quantitative data on several hundred *T. gondii* tachyzoites in a single field of view, and provides new opportunities to study these and other questions that are key to understanding the mechanisms underlying *T. gondii* motility within infected hosts.

## Supporting Information

Figure S1 **Motility analysis of heat-killed and cytochalasin D-treated parasites.** (**A**) Representative MIP of parasites that were heat-killed by incubating at 56°C for 30 min prior to adding to the Pitta chamber and imaging. Scale bar = 50 µm. (**B**) Histogram of trajectory displacements for the heat-killed parasite preparation. 97.6% of heat-killed parasites had a trajectory displacement of 2 µm or less, with the remaining trajectories the result of mistracking artifacts. Results shown are representative of two independent experiments. (**C**) Representative MIP of parasites that were pretreated for 15 min at 37°C with 0.2 µM cytochalasin D prior to adding to the Pitta chamber and imaging. Scale bar = 50 µm. (**D**) Histogram of trajectory displacements for the cytochalasin D-treated parasite preparation. 89.9% of cytochalasin D-treated parasites had a trajectory displacement of 2 µm or less, with the remaining trajectories the result of mistracking artifacts. Results shown are representative of two independent experiments. The colour scheme for all MIPs was inverted for better visualization of parasite trajectories.
(TIF)

Figure S2 **PDB file of the raw (unsmoothed) trajectory from** **Figure 2A** **.** The .pdb file can be viewed using molecular graphics software such as Jmol [55] or PyMOL [28], [29].
(PDB)

Figure S3 **PDB file of the smoothed trajectory from** **Figure 2A** **.** The .pdb file can be viewed using molecular graphics software such as Jmol [55] or PyMOL [28], [29].
(PDB)

Figure S4 **PDB file of the raw (unsmoothed) trajectory from** **Figure 2B** **.** The .pdb file can be viewed using molecular graphics software such as Jmol [55] or PyMOL [28], [29].
(PDB)

Figure S5 **PDB file of the smoothed trajectory from** **Figure 2B** **.** The .pdb file can be viewed using molecular graphics software such as Jmol [55] or PyMOL [28], [29].
(PDB)

Figure S6 **A schematic description of curvature.** (**A**) Intuitively, curvature can be thought of as the degree to which the curve bends within a plane at a given point **p**, per unit of contour length (distance along the curve). The discrete approximation to curvature is given by the exterior angle **θ** at **p** between two nearby points **a** and **b** on the curve; *i.e.*, angle **bpc**, where **c** is simply an extension of the line **ap**. (**B**) As points **a** and **b** are brought closer along the curve to point **p**, the curvature of a circle passing through **a** **b** **p** more closely matches the curvature of the curve at point **p**; in the limit, the true curvature of the curve is identical to that of this osculating circle. Since the curvature of a circle is the reciprocal of its radius, the instantaneous curvature of the curve at point **p** is **κ**. A greater bend in the curve at a given point gives rise to an osculating circle with a smaller radius, and hence has a greater curvature at this point.
(TIF)

Figure S7 **A schematic description of torsion.** (**A**) Torsion is a measure of the extent to which the curve twists out of the plane at a given point **p**, per unit of contour length. The discrete approximation to torsion is given by the dihedral angle **φ** through points **a** **b** **c** and **d** on the curve near **p**; *i.e.*, the angle between the two planes defined by points **a** **b** **c** and points **b** **c** **d** as viewed down the line segment **bc**. This angle is signed, and is measured from the plane defined by points **a** **b** **c** to the plane defined by points **b** **c** **d** as viewed down the line segment **bc**. (**B**) As points **a** **b** **c** and **d** are brought closer along the curve to point **p**, we can devise a sphere, analogous to the osculating circle described in Figure S6, that is tangent to both planes defined by points **a** **b** **c** and points **b** **c** **d**. In the limit, the instantaneous torsion of the curve at point **p** is given by **τ**, the reciprocal of the radius of this sphere. A greater twist magnitude between these two adjacent bends, **a** **b** **c** and **b** **c** **d**, gives rise to a sphere with smaller radius, and hence has a larger torsion magnitude at point **p**.
(TIF)

Figure S8 **Differential interference contrast images illustrating morphology differences between RH, Δ** ***phil1*** **and Comp parasites.** Scale bar = 5 µm. Images were adapted from [22].
(TIF)

Video S1 ***ex vivo*** **motility of RH-OVA-tdTomato** ***T. gondii*** **parasites from** **Figure 1A** **.** RH strain *T. gondii* tachyzoites expressing a tandem tomato fluorescence cassette (RH-OVA-tdTomato) were injected into a mouse earflap and imaged by two-photon laser scanning microscopy. Parasites move in a corkscrew-like fashion, often with pauses in between each unit of movement. Video playback is set at 31× speed. Scale bar = 29 µm.
(MOV)

Video S2 **Trail deposition of parasites in Matrigel.** Parasites were incubated with anti-TgSAG1 conjugated to Alexa Fluor 488, resuspended in diluted Matrigel, and imaged every 0.88 s for 60 s by time-lapse fluorescence videomicroscopy. Parasites can be seen depositing TgSAG1 as they undergo corkscrew-like trajectories in Matrigel. Video playback is set at 8.8× speed. Scale bar = 10 µm.
(MOV)

Video S3 **Motility of RH parasites in Matrigel from** **Figure 1D** **.** RH parasites were resuspended in diluted Matrigel containing Hoechst 33342. Fluorescent parasite nuclei were imaged in a 402 µm×401 µm×40 µm volume (1 µm z-slices) every 0.88 s for 60 s by time-lapse fluorescence videomicroscopy. Parasites move in left-handed, corkscrew-like trajectories in Matrigel; one representative trajectory is highlighted in green. Video playback is set at 4.5× speed. Scale bar = 10 µm.
(MOV)
