# Supplementary figures and images for: Disruption of TgPHIL1 Alters Specific Parameters of Toxoplasma gondii Motility Measured in a Quantitative, Three-Dimensional Live Motility Assay
Source: PLoS One. 2014 Jan 29;9(1):e85763. doi: 10.1371/journal.pone.0085763 (PMC3906025; doi:10.1371/journal.pone.0085763)

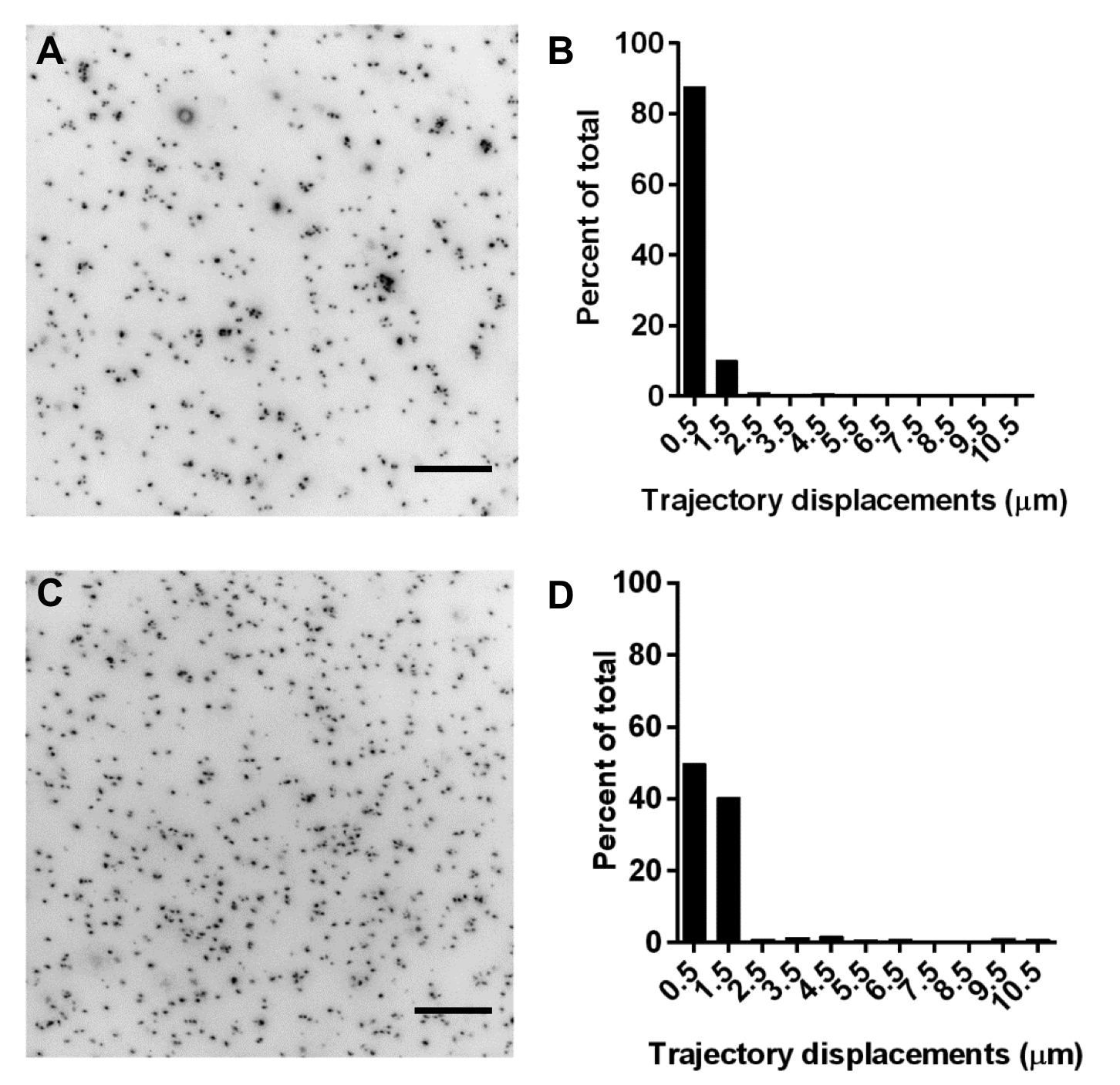

Supplement: Figure S1 — Motility analysis of heat-killed and cytochalasin D-treated parasites. (A) Representative MIP of parasites that were heat-killed by incubating at 56°C for 30 min prior to adding to the Pitta chamber and imaging. Scale bar = 50 µm. (B) Histogram of trajectory displacements for the heat-killed parasite preparation. 97.6% of heat-killed parasites had a trajectory displacement of 2 µm or less, with the remaining trajectories the result of mistracking artifacts. Results shown are representative of two independent experiments. (C) Representative MIP of parasites that were pretreated for 15 min at 37°C with 0.2 µM cytochalasin D prior to adding to the Pitta chamber and imaging. Scale bar = 50 µm. (D) Histogram of trajectory displacements for the cytochalasin D-treated parasite preparation. 89.9% of cytochalasin D-treated parasites had a trajectory displacement of 2 µm or less, with the remaining trajectories the result of mistracking artifacts. Results shown are representative of two independent experiments. The colour scheme for all MIPs was inverted for better visualization of parasite trajectories. (TIF) [file pone.0085763.s001.tif]

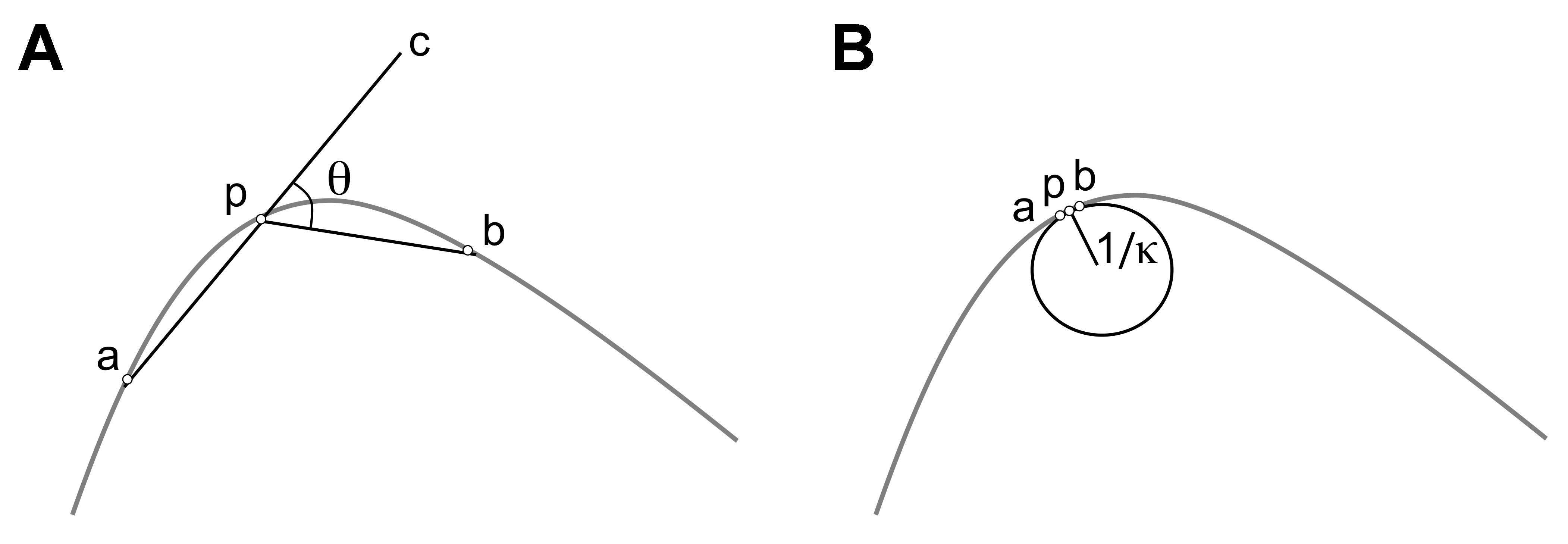

Supplement: Figure S6 — A schematic description of curvature. (A) Intuitively, curvature can be thought of as the degree to which the curve bends within a plane at a given point p, per unit of contour length (distance along the curve). The discrete approximation to curvature is given by the exterior angle θ at p between two nearby points a and b on the curve; i.e., angle bpc, where c is simply an extension of the line ap. (B) As points a and b are brought closer along the curve to point p, the curvature of a circle passing through a b p more closely matches the curvature of the curve at point p; in the limit, the true curvature of the curve is identical to that of this osculating circle. Since the curvature of a circle is the reciprocal of its radius, the instantaneous curvature of the curve at point p is κ. A greater bend in the curve at a given point gives rise to an osculating circle with a smaller radius, and hence has a greater curvature at this point. (TIF) [file pone.0085763.s006.tif]

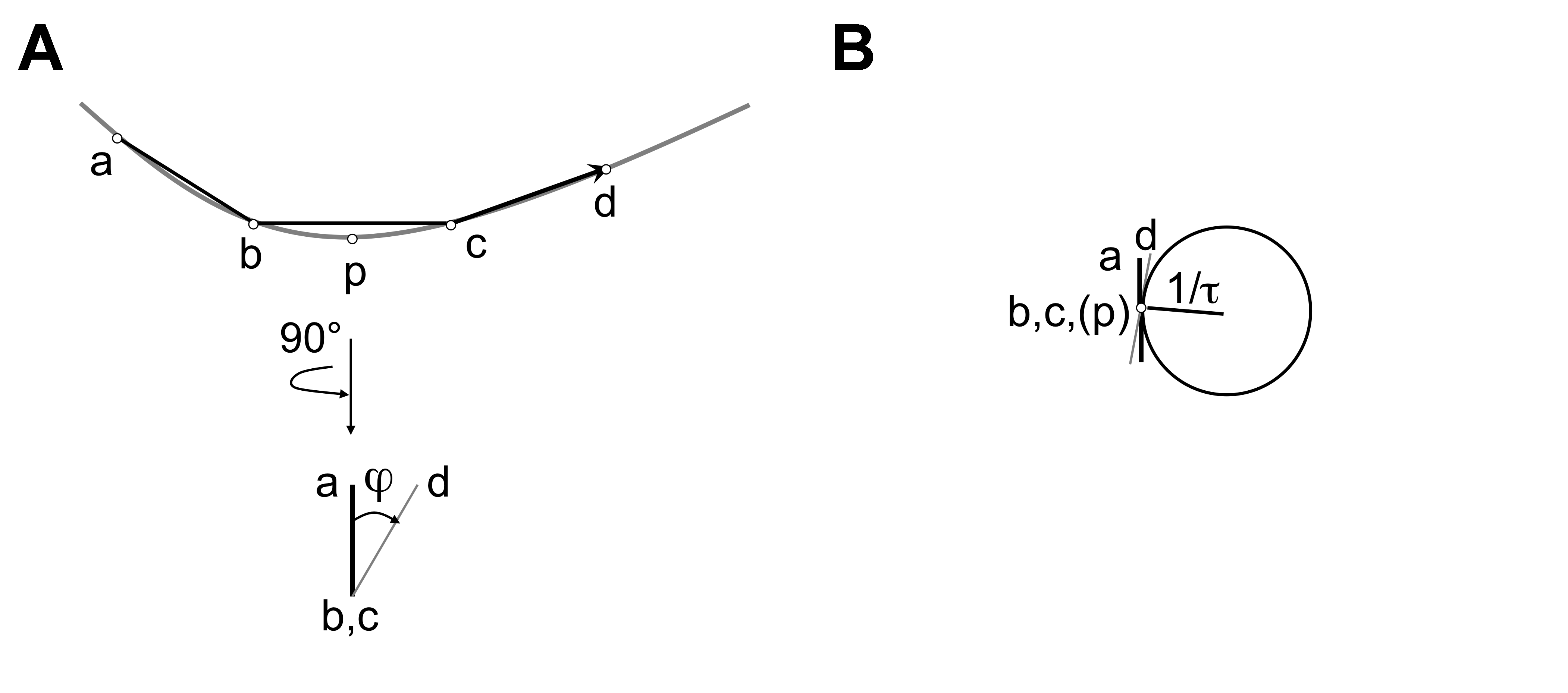

Supplement: Figure S7 — A schematic description of torsion. (A) Torsion is a measure of the extent to which the curve twists out of the plane at a given point p, per unit of contour length. The discrete approximation to torsion is given by the dihedral angle φ through points a b c and d on the curve near p; i.e., the angle between the two planes defined by points a b c and points b c d as viewed down the line segment bc. This angle is signed, and is measured from the plane defined by points a b c to the plane defined by points b c d as viewed down the line segment bc. (B) As points a b c and d are brought closer along the curve to point p, we can devise a sphere, analogous to the osculating circle described in Figure S6, that is tangent to both planes defined by points a b c and points b c d. In the limit, the instantaneous torsion of the curve at point p is given by τ, the reciprocal of the radius of this sphere. A greater twist magnitude between these two adjacent bends, a b c and b c d, gives rise to a sphere with smaller radius, and hence has a larger torsion magnitude at point p. (TIF) [file pone.0085763.s007.tif]

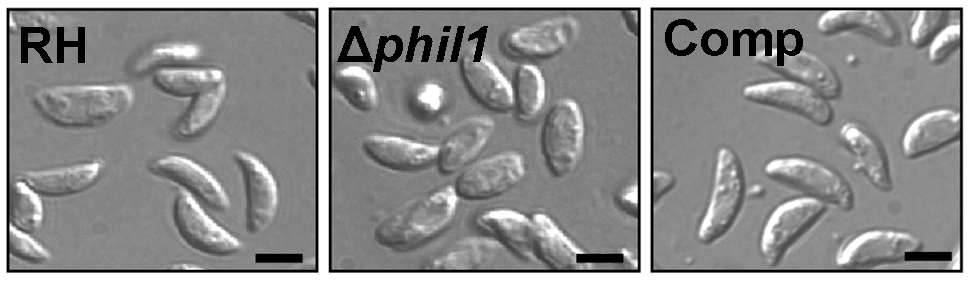

Supplement: Figure S8 — Differential interference contrast images illustrating morphology differences between RH, Δ phil1 and Comp parasites. Scale bar = 5 µm. Images were adapted from [22]. (TIF) [file pone.0085763.s008.tif]
